# A Systematic Review of Kernel-Level Security Mechanisms, Vulnerability Detection and Mitigation in Modern Operating Systems

**DOI:** 10.3390/s26082452

**Published:** 2026-04-16

**Authors:** Zeeshan Ali, Naeem Aslam, Andrea Marotta, Walter Tiberti, Dajana Cassioli

**Affiliations:** 1 Department of Information Engineering Computer Science and Mathematics, University of L’Aquila, 67100 L’Aquila, Italy; andrea.marotta@univaq.it (A.M.); walter.tiberti@univaq.it (W.T.); dajana.cassioli@univaq.it (D.C.); 2 Department of Computer Science, National College of Business Administration and Economics, Lahore 54660, Pakistan; naeemaslam247@gmail.com

**Keywords:** operating systems, vulnerability detection, kernel security, exploit mitigation, secure boot, kernel integrity

## Abstract

Kernel attacks are still one of the most severe threats to modern operating systems (OS) due to the kernel’s privileged control over hardware, memory, and process management. This study reviews some significant kernel-level security mechanisms regarding vulnerability detection, as well as the prevention and mitigation of exploitation in today’s OSs. Using the Preferred Reporting Items for Systematic Reviews and Meta-Analyses (PRISMA) methodology, a total of 30 high-quality, peer-reviewed studies were examined and analyzed in detail using the Critical Appraisal Skills Programme (CASP) quality framework. Discussion about the leading research directions emanated from three central questions of this review: What are the predominant kernel attack vectors? How are the techniques for protection and detection that are currently available assessed? What are the emerging research directions? The study identifies the following as the principal sources of kernel compromise: memory corruption, privilege escalation, rootkits, and race condition exploits. It also identifies several techniques for kernel hardening, such as Mandatory Access Control (MAC), the use of SELinux and AppArmor, kernel integrity monitoring, secure and measured boot, fuzz testing, and hardware-assisted protection. Some of these emerged as having a great deal of promise for proactive defense against zero-day vulnerabilities, including machine learning-based detection and live kernel patching. Issues regarding scalability, detection accuracy, and securing containerized and virtualized environments need to be solved. This paper aims to provide relevant, structured, and up-to-date research on kernel security synthesis and offer valuable guidance on the development of robust, adaptive, and novel OS defense mechanisms.

## 1. Introduction

Modern operating systems (OSs) rely on the kernel to manage memory, processes, devices, and privilege boundaries. Because the kernel executes with the highest privilege level, a successful kernel compromise can invalidate all higher-layer protections and enable persistent control over the platform. Therefore, rootkits, privilege-escalation exploits, race-condition abuse, and memory-corruption attacks remain central concerns in operating system security research.

Contemporary platforms including Linux, Windows, Android, macOS, and Unix-derived systems increasingly deploy kernel-focused protections such as secure and measured boot, kernel address randomization, integrity monitoring, mandatory access control, and hardware-assisted isolation [1,2,3,4]. At the same time, incidents such as Dirty COW, Meltdown, Spectre, and BlueKeep demonstrate that kernel attack surfaces continue to evolve faster than any single defense mechanism can address [5,6]. As a result, vulnerability discovery and mitigation now depend on a combination of static analysis, runtime monitoring, integrity verification, fuzzing, and targeted hardening measures [7,8,9].

The need for a new systematic review follows from a gap in how the literature is currently organized. Existing review papers tend either to discuss operating system security at a broad level or to focus on narrower topics such as machine learning-assisted vulnerability discovery [9,10]. This study differs by systematically synthesizing prevention, detection, and mitigation mechanisms at the kernel level under an explicit PRISMA-based selection workflow and a CASP-inspired quality screening process for peer-reviewed studies published between 2010 and 2024. The objective is not only to summarize mechanisms but also to clarify how the reviewed papers support the resulting conclusions and where the evidence remains incomplete.

**Figure 1:**
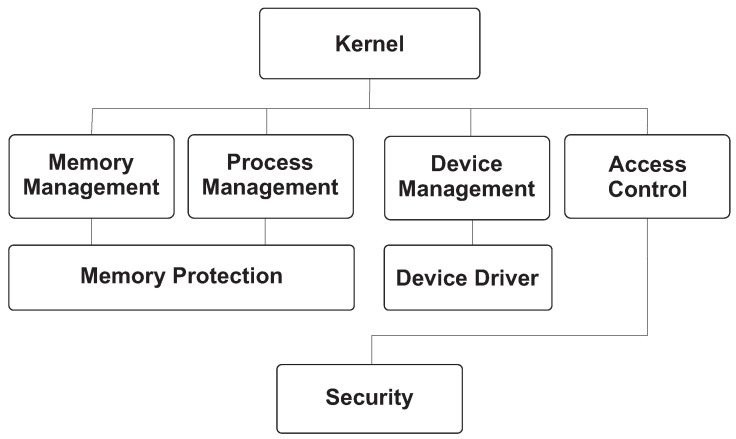
General architecture of an OS kernel.

**Figure 2:**
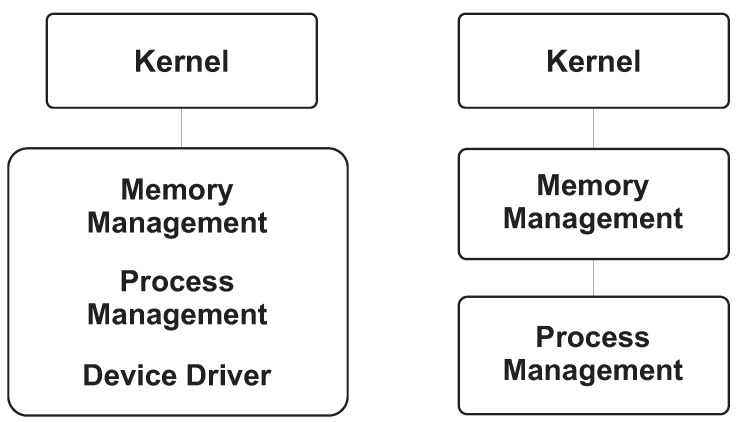
Structural comparison of a microkernel and monolithic kernel.

**Figure 3:**
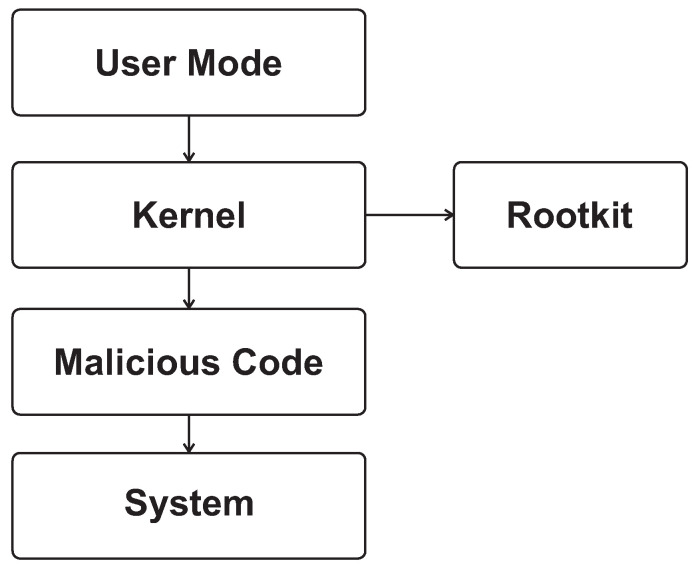
Kernel rootkit infiltration process.

**Figure 4:**
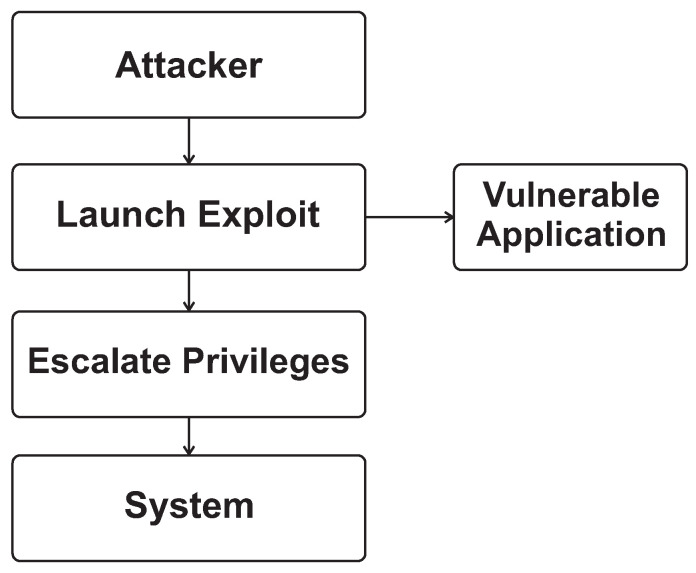
Privilege escalation attack model.

**Figure 5:**
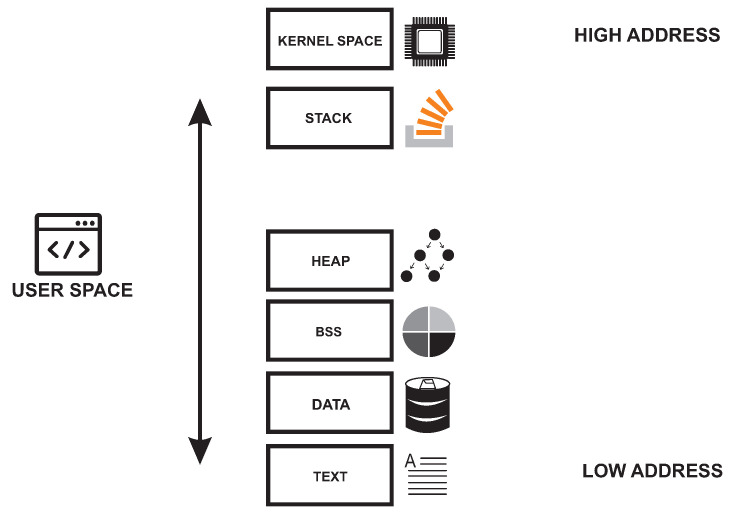
Kernel buffer overflow exploitation stages.

**Figure 6:**
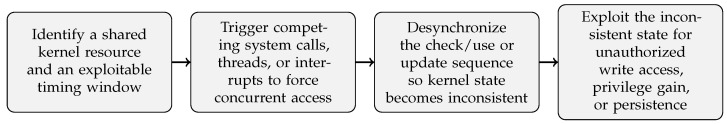
Generalized kernel race-condition exploitation sequence synthesized for this review.

Figure 1 and Figure 2 provide the architectural context for this review by illustrating the main OS kernel components and the distinction between monolithic and microkernel designs. Figure 3, Figure 4, Figure 5 and Figure 6 then summarize representative attack vectors that motivate the reviewed defenses.

Despite significant advances in kernel protection, contemporary OSs remain susceptible to complex, focused attacks that take advantage of kernel vulnerabilities, ineffective access controls, inefficient memory protection, or out-of-date code. Existing security mechanisms also fall short in view of emerging threats like zero-day kernel vulnerabilities, hardware-assisted attacks, kernel rootkits, and privilege escalation exploits. The literature lacks any consolidated knowledge regarding strengths, weaknesses, and relative efficacy among current kernel-level security mechanisms.

Figure 3, Figure 4, Figure 5 and Figure 6 summarize representative kernel-level attack vectors that motivate the reviewed defenses. Figure 3 illustrates a rootkit infiltration process in which malicious code first reaches the system through a vulnerable kernel-facing entry point such as a driver, loadable module, or privileged execution path, then embeds itself inside the kernel; modifies kernel behavior; and attempts to remain hidden by concealing processes, files, network activity, or hooks. The figure therefore emphasizes that the main danger of kernel rootkits is not only initial compromise but also stealth, persistence, and the ability to tamper with core OS services after installation.

Figure 4 depicts a privilege-escalation attack model in which an attacker starts from a lower-privilege context, abuses a weakness in a kernel interface or privileged component, crosses the user–kernel trust boundary, and eventually obtains elevated control over protected resources. The figure is intended to show the stepwise transition from limited user-space access to administrative or kernel-level authority, highlighting how a single flaw in privilege enforcement can cascade into unrestricted access to sensitive memory, system configuration, and security controls.

Figure 5 presents the main stages of kernel buffer-overflow exploitation. It shows how an oversized or improperly validated input can overwrite adjacent kernel memory, corrupt control data or critical objects, redirect execution, and ultimately enable arbitrary code execution or policy bypass in a privileged context. The figure is included to clarify that memory-corruption attacks are dangerous not merely because a buffer is overrun but because the corruption can be transformed into control-flow hijacking, integrity compromise, and system-wide privilege abuse.

Figure 6 is intentionally a conceptual synthesis rather than the architecture of a specific framework. It abstracts the common stages of kernel race-condition exploitation into (i) identification of a shared resource and an exploitable timing window; (ii) forcing of concurrent access through competing system calls, threads, or interrupts; (iii) induction of an inconsistent check/use or update sequence; and (iv) leveraging of the resulting inconsistency to obtain unauthorized state changes, privilege escalation, or persistent control. This framing reflects the generic exploitation logic discussed in the kernel race-vulnerability literature rather than any single implementation-specific workflow [11].

Most earlier works analyzed individual kernel security mechanisms or presented non-systematic reviews of selected techniques. However, there is limited systematic research that synthesizes all the major kernel-level security approaches that focus simultaneously on prevention, detection, and mitigation. A comparison of various mechanisms that different OSs implement to provide security, remaining gaps in vulnerabilities, and the effectiveness of various detection tools against new attack vectors has not been conducted.

Because kernel-level attacks threaten how safe, private, and accessible systems remain, studying them matters. From different angles, experts see patterns when they take time to analyze past findings carefully. Seeing gaps in today’s defenses opens space for better solutions over time. What emerges shapes both classroom ideas and real-world OSs alike.

This systematic review has two key objectives: to present a comprehensive study of kernel-level security mechanisms and to evaluate vulnerability detection and mitigation approaches used in modern OSs.

Research objectives include the following:

- To identify and classify kernel-level security mechanisms within modern OSs;
- To assess the effectiveness of kernel-level vulnerability detection methods;
- To analyze the mitigation strategies used to prevent kernel exploitation;
- To indicate limitations of research and suggest further research.

### Research Questions

RQ1: What are the main kernel-level security mechanisms implemented in modern OSs?
RQ1.1: How do access control frameworks contribute to kernel protection?
RQ1.2: What memory isolation and integrity techniques defend against kernel attacks?
RQ1.3: How effective are hardware-assisted kernel security features?
RQ2: What vulnerability detection techniques are used at the kernel level?
RQ2.1: What static analysis tools detect kernel vulnerabilities?
RQ2.2: What dynamic or runtime monitoring techniques are used?
RQ3: What mitigation strategies are used to protect kernels from exploitation?
RQ3.1: How effective are exploit mitigation frameworks such as Address Space Layout Randomization (ASLR) and Data Execution Prevention (DEP)?
RQ3.2: What are the limitations of existing mitigation techniques?
RQ3.3: What future enhancements are proposed for kernel security?

The motivations corresponding to each research question are summarized in Table 1.

## 2. Literature Review

Kernel security research spans policy enforcement, memory isolation, integrity assurance, exploit mitigation, and vulnerability discovery. Broad overviews of operating system security identified kernel compromise as a recurring root cause of systemic failures, while later studies narrowed attention to specific hardening and attack-surface reduction mechanisms [3,4,10]. This progression shows a shift from high-level threat categorization toward mechanism-specific evaluation.

Access control and isolation studies have emphasized limiting privileged interactions inside the kernel. Attribute-based and policy-driven controls extend traditional mandatory access control models, while container-aware kernel mechanisms use Linux security modules, namespaces, and control groups to constrain kernel-mediated access to sensitive resources [12,13,14]. These works consistently report that finer-grained policy enforcement improves containment, although deployment complexity and policy maintenance remain practical barriers.

Additional kernel-focused studies also examine peripheral and hardware debug attack surfaces in deployed systems, further highlighting the need for policy-aware device mediation and platform-specific hardening in Linux and ARM ecosystems [15,16].

Memory protection and hardware-assisted defenses form another major stream of the literature. Kernel address randomization and attack-surface reduction raise the cost of code-reuse and privilege-escalation exploits, but microarchitectural attacks demonstrate that software-only hardening remains insufficient under adversaries with side-channel capabilities [1,2,4,17]. The reviewed evidence therefore supports defense-in-depth rather than single-mechanism protection.

The vulnerability detection literature is dominated by fuzzing, runtime integrity monitoring, and hybrid analysis. Guided kernel fuzzing improves coverage and bug discovery, trusted execution-based runtime protection strengthens post-deployment monitoring, and recent reviews highlight growing interest in learning-assisted techniques for vulnerability discovery and prioritization [7,8,9]. At the same time, the literature remains uneven in evaluation practices, with many studies reporting different metrics, platforms, and workloads.

Race condition-oriented kernel fuzzing further reinforces this trend by demonstrating that concurrency-aware fuzzing can expose high-impact kernel bugs not captured by conventional coverage-only strategies [11].

### 2.1. Kernel-Level Security Mechanisms in Modern Operating Systems

**Figure 7:**
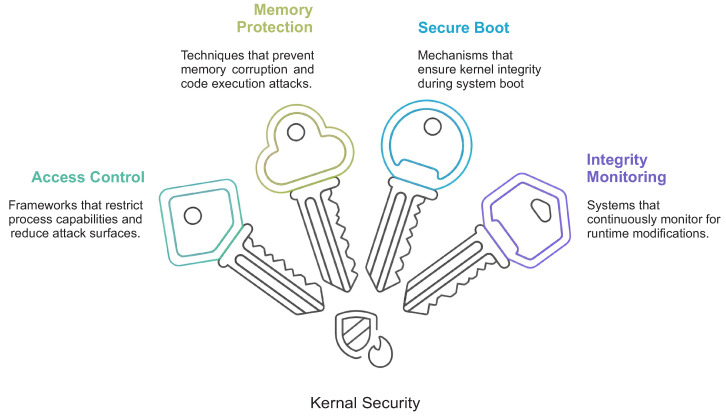
Kernel-level security framework.

The reviewed studies show that kernel-level security mechanisms are usually organized as layered protections rather than isolated controls. The dominant categories are mandatory access control, memory isolation and layout diversification, secure boot and integrity enforcement, kernel attack-surface reduction, and hardware-assisted isolation [1,3,4,12,17]. Figure 7 summarizes these categories and their relationship to privileged attack surfaces.

Mandatory access control frameworks such as SELinux- and AppArmor-style policy enforcement remain important because they reduce the impact of privilege misuse inside the kernel boundary. More recent work extends this direction with attribute-based policies and container-aware controls to support dynamic workloads and multi-tenant environments [12,13]. These mechanisms strengthen policy enforcement, but they also depend on correct policy design and sustained administrative effort.

Memory protection mechanisms such as kernel address space layout randomization (KASLR), supervisor-mode execution restrictions, and attack-surface reduction techniques aim to make kernel exploitation less reliable [4,17]. However, studies of Meltdown-class attacks demonstrate that microarchitectural leakage can weaken otherwise robust software defenses [2]. This explains why the literature increasingly combines software hardening with hardware-aware isolation and execution controls [1].

Secure and measured boot, module signing, and runtime integrity verification provide assurance that the executing kernel has not been tampered with before or during execution [3,8]. These mechanisms do not eliminate vulnerabilities by themselves, but they improve resilience against persistence-oriented attacks by narrowing opportunities for unauthorized kernel modification.

### 2.2. Vulnerability Detection Techniques at the Kernel Level

Detecting kernel vulnerabilities remains harder than application-level testing because kernel code executes with privileged access to hardware, memory management, and synchronization primitives. Across the reviewed studies, the main detection families are static analysis, dynamic or runtime monitoring, and fuzzing-based discovery [7,8,9,10].

Static analysis supports early-stage vulnerability discovery by examining kernel source code without execution. The reviewed literature associates static analysis with the discovery of memory-safety issues, race conditions, and logic flaws but also reports recurring false-positive and scalability limitations in large kernels [9,10]. For this reason, static techniques are most effective when combined with complementary runtime validation.

**Figure 8:**
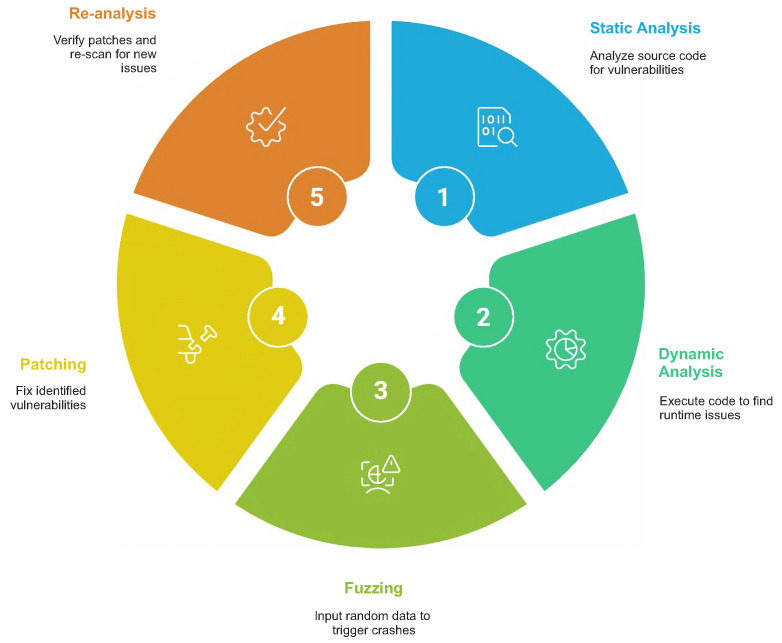
Vulnerability detection cycle.

Dynamic and runtime monitoring techniques observe kernel behavior during execution through instrumentation, integrity verification, or trusted execution-based protection. These approaches improve fidelity when detecting real attack conditions, but the reviewed studies also note non-trivial runtime overhead and evasion challenges [3,8]. Figure 8 outlines the resulting vulnerability detection cycle.

Kernel fuzzing is the most prominent vulnerability discovery strategy in the reviewed literature. Guided syscall fuzzing and related hybrid approaches achieve strong coverage and repeatedly expose previously unknown bugs, especially memory-corruption vulnerabilities [7,9]. Learning-based support appears in this literature, mainly as a way to prioritize inputs, increase coverage, or classify discovered behaviors rather than as a single dominant artificial intelligence approach.

### 2.3. Kernel-Level Vulnerability Mitigation and Exploit Prevention

Kernel-level mitigation strategies are designed to limit exploit success, reduce the exposed attack surface, and contain the damage caused by successful compromise attempts. The reviewed literature repeatedly highlights memory-execution controls, privilege separation, attack-surface reduction, live patching, and hardware-assisted enforcement as the main mitigation families [1,2,3,4,14].

Address Space Layout Randomization (ASLR) and Data Execution Prevention (DEP) remain foundational exploit mitigation mechanisms because they disrupt code injection and increase the difficulty of reliable code-reuse attacks. Nevertheless, the reviewed studies show that side channels, information leaks, and return-oriented programming can still weaken these defenses if they are deployed in isolation [2,4].

Privilege separation, intra-kernel sandboxing, and driver isolation reduce the blast radius of vulnerable kernel components by limiting direct interaction with the most sensitive execution domains [13,14]. These mechanisms improve containment but introduce engineering and compatibility tradeoffs that affect real-world deployment.

**Figure 9:**
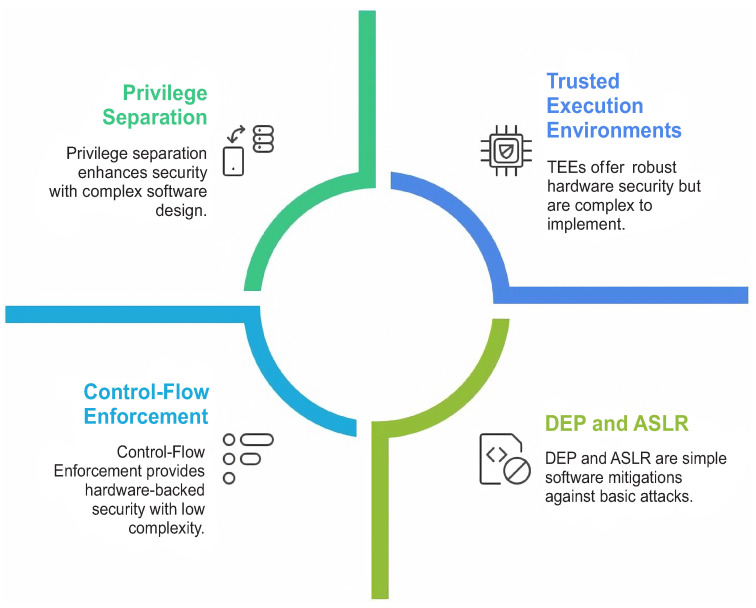
Kernel-level vulnerability mitigation techniques.

Hardware-assisted protection and live update techniques aim to increase resilience without depending exclusively on software checks. In the reviewed literature, hardware-supported isolation is reported to improve protection against advanced attacks, whereas operational techniques such as live patching are reported to improve availability during remediation. Figure 9 summarizes the principal mitigation categories discussed in the reviewed studies [1,3].

### 2.4. Limitations of Existing Kernel-Level Security Approaches in the Literature

Despite clear progress, the reviewed literature shows several persistent limitations. Many mechanisms remain reactive and are evaluated primarily against known exploit classes rather than against previously unseen attack combinations [9,10]. Performance overhead, deployment complexity, and policy misconfiguration also remain recurring practical concerns, particularly for runtime monitoring and fine-grained policy enforcement [8,13].

**Figure 10:**
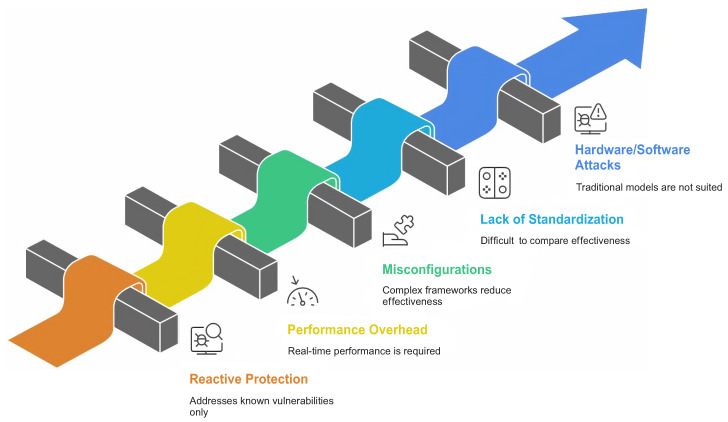
Kernel security limitations.

The literature is also fragmented across platforms and evaluation methods. Linux-focused studies dominate the corpus, while Windows- and macOS-specific kernel defenses appear less frequently. This makes direct cross-platform comparison difficult and limits the generalizability of some conclusions. Figure 10 summarizes the main limitations repeatedly reported in the reviewed studies.

### 2.5. Summary of Literature Review

The literature review shows that kernel security research has converged on a defense-in-depth model combining policy enforcement, memory and execution isolation, integrity verification, fuzzing, and exploit mitigation. At the same time, the reviewed studies reveal persistent gaps in cross-platform coverage, evaluation consistency, and support for emerging containerized and hardware-assisted attack scenarios. These observations directly motivate the methodological design and research questions of this review.

## 3. Methodology

### 3.1. Search Strategy

The search strategy was designed to identify peer-reviewed studies addressing kernel-level security mechanisms, vulnerability detection, and exploit mitigation in modern operating systems. Five academic databases were queried, as shown in the Table 2: Taylor & Francis Online, ACM Digital Library, ScienceDirect, Sage Journals, and IEEE Xplore. The search period was limited to 2010–2024, and only English-language journal and conference papers were considered.

**Figure 11:**
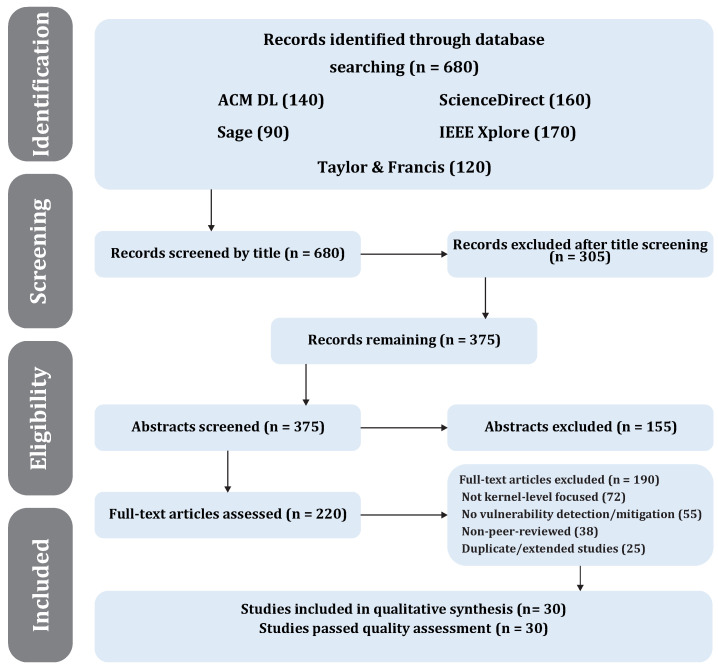
PRISMA flow diagram of study selection.

The same conceptual search blocks were preserved across all databases: kernel security, vulnerability detection, exploit mitigation, and operating systems. The wording of individual queries was adjusted only to satisfy database-specific search syntax. After retrieval, the candidate records were screened by title, abstract, and full text, and the resulting workflow is documented in the PRISMA diagram shown in Figure 11.

### 3.2. Selection Strategy

After the initial search, records were screened in five stages: keyword retrieval, title screening, abstract screening, introduction and conclusion screening, and full-text eligibility assessment as shown in the Table 3. A study was retained only when it addressed kernel-level protection, vulnerability detection, or mitigation in modern operating systems and provided sufficient methodological or technical detail to support qualitative synthesis. Figure 11 summarizes the resulting study-selection workflow.

### 3.3. Inclusion Criteria

The following criteria were applied to include relevant studies in this systematic review:

- Peer-reviewed journal articles or conference papers;
- Publications released between 2010 and 2024;
- Studies written in English.
- Studies focused on kernel-level security mechanisms, kernel vulnerability detection, or kernel exploit mitigation in modern operating systems;
- Studies providing technical, experimental, comparative, or systematic evidence relevant to the research questions;
- Full-text articles available through open access or institutional library access.

### 3.4. Exclusion Criteria

The following exclusion criteria were applied to remove irrelevant studies:

- Studies focusing only on user-level or application-level security without kernel involvement;
- Papers without full-text availability;
- Non-peer-reviewed publications such as blogs, white papers, reports, tutorials, preprints, and archive-only manuscripts;
- Duplicate studies retrieved from multiple databases;
- Research not directly related to kernel security, vulnerability detection, or mitigation techniques;
- Studies published before 2010;
- Studies published after 2024;
- Papers not written in the English language.

### 3.5. Quality Assessment

Table 4 summarizes the quality screening applied to the eligible studies. We adopted a binary CASP-inspired scoring scheme to reduce ambiguity during study selection and to preserve consistent inclusion decisions across heterogeneous study designs. Each paper was assessed against five criteria: clarity of objectives, methodological validity, direct kernel-security relevance, experimental validation, and research contribution. Each criterion was scored as yes (1) or no (0), and studies scoring at least 4 out of 5 were retained for synthesis. Because the binary model is intended for admissibility screening rather than fine-grained ranking, the final interpretation of the evidence remains qualitative and is discussed narratively in Section 5.

For traceability, the 30 accepted studies correspond to the study identifiers in Table 4 as follows: S1 [18], S2 [12], S3 [1], S4 [19], S5 [2], S6 [20], S7 [7], S8 [17], S9 [21], S10 [22], S11 [8], S12 [13], S13 [14], S14 [9], S15 [23], S16 [24], S17 [25], S18 [26], S19 [27], S20 [3], S21 [28], S22 [29], S23 [4], S24 [30], S25 [31], S26 [32], S27 [10], S28 [33], S29 [34], and S30 [35].

The S1–S30 set above constitutes the primary evidence corpus used for PRISMA-based synthesis. Additional citations included elsewhere are contextual background references and are not counted as part of the screened study set.

To strengthen reproducibility, this revision is accompanied by a supplementary study-traceability package prepared for repository-based sharing available at [36]. The package records the full included corpus (S1–S30) with persistent paper links. It also provides the database search strings and a search-rerun candidate list derived from those queries within the same 2010–2024 review window to support independent re-execution of the retrieval step. Because the original platform-level export of excluded records was not preserved during screening, the repository material should be interpreted as a traceability and rerun aid rather than as a verbatim exclusion log.

## 4. Results

This section presents the synthesized findings derived from the qualitative analysis of the 30 selected studies. Although methodological diversity and heterogeneous evaluation metrics limited direct quantitative comparison, clear patterns emerged regarding dominant kernel-level security mechanisms, vulnerability detection techniques, and mitigation strategies. The results demonstrate consistent alignment with the research questions (RQ1–RQ3) while also revealing structural gaps in evaluation practices and cross-platform generalizability.

All selected studies satisfied the predefined quality threshold (Table 4), indicating that the included literature demonstrates clear objectives, sufficient methodological transparency, and direct relevance to kernel-level security. Figure 11 provides the underlying selection traceability for these results.

### 4.1. RQ1: Kernel-Level Security Mechanisms in Modern OSs

The analysis of the selected studies indicates that modern OSs adopt a layered defense strategy at the kernel level to mitigate privileged attacks and reduce systemic vulnerabilities. Across the reviewed literature, five dominant categories of kernel-level security mechanisms were consistently identified:

- MAC frameworks (e.g., SELinux and AppArmor);
- Memory protection mechanisms (e.g., KASLR, SMEP, and SMAP);
- Secure and measured boot architectures;
- Kernel integrity monitoring systems;
- Hardware-assisted security mechanisms.

**Figure 12:**
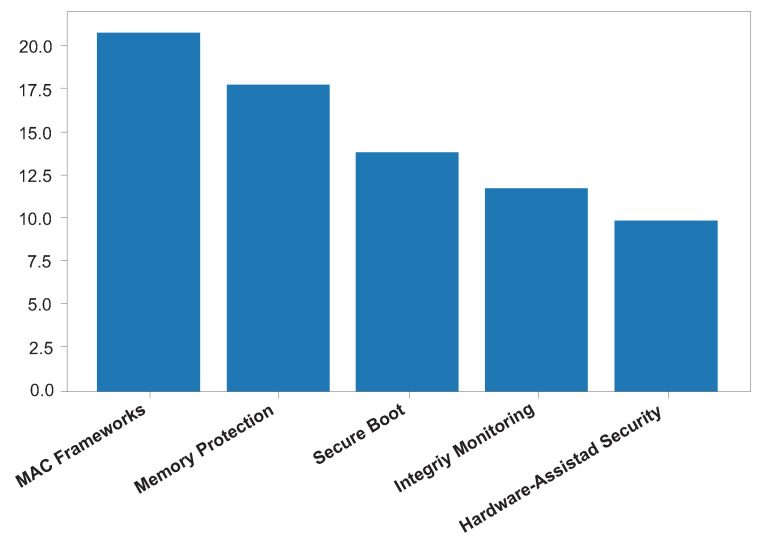
Distribution of kernel-level security mechanisms identified in the reviewed studies.

The distribution of kernel-level security mechanisms across the reviewed studies (Figure 12) reveals that mandatory access control frameworks and memory-protection techniques are the most frequently reported safeguards [4,12,13,17]. This dominance highlights the continued reliance on policy enforcement and memory isolation as foundational pillars of kernel security.

Mandatory access control frameworks play a critical role in restricting unauthorized privilege escalation by enforcing fine-grained security policies within the kernel [12,13]. Memory protection mechanisms such as KASLR, Supervisor Mode Execution Prevention (SMEP), and Supervisor Mode Access Prevention (SMAP) further enhance resilience against memory corruption and code-reuse attacks by limiting predictable memory layouts and restricting illegitimate memory access [4,17].

Secure boot and kernel integrity monitoring mechanisms exhibit moderate representation in the literature. Their presence underscores their importance as complementary safeguards that ensure kernel trustworthiness during system initialization and runtime [3,8]. These mechanisms primarily focus on detecting or containing unauthorized kernel modification rather than eliminating initial exploitation opportunities.

In contrast, hardware-assisted security mechanisms appear less frequently, despite their increasing relevance. This limited representation may be attributed to their platform dependency, deployment complexity, and the smaller number of peer-reviewed studies within the review window. Nevertheless, the reviewed studies emphasize that hardware-supported defenses provide stronger isolation guarantees and improved protection against advanced attack vectors [1,2].

Overall, the findings suggest that contemporary kernel security architectures prioritize software-enforced controls, while hardware-assisted protections are emerging as a critical enhancement layer for future OS designs.

Table 5 further illustrates that Linux-based kernel security mechanisms dominate the literature, while comparatively fewer peer-reviewed studies investigate Windows and macOS kernel defenses within the same review window. This imbalance suggests a clear research gap in cross-platform kernel security evaluation.

### 4.2. RQ2: Kernel-Level Vulnerability Detection Techniques

The review identifies three dominant categories of kernel-level vulnerability detection techniques consistently discussed across the selected studies:

- Static Analysis: Early detection of vulnerabilities through examination of kernel source code without execution;
- Dynamic Analysis: Runtime monitoring, instrumentation, and integrity verification;
- Fuzzing-Based Detection: Automated discovery of vulnerabilities via input mutation and fault triggering.

Among these approaches, fuzzing-based detection emerges as the most impactful technique. The reviewed studies consistently describe guided or hybrid kernel fuzzing as highly effective for the discovery of memory-corruption flaws and previously unknown vulnerabilities [7,9].

**Figure 13:**
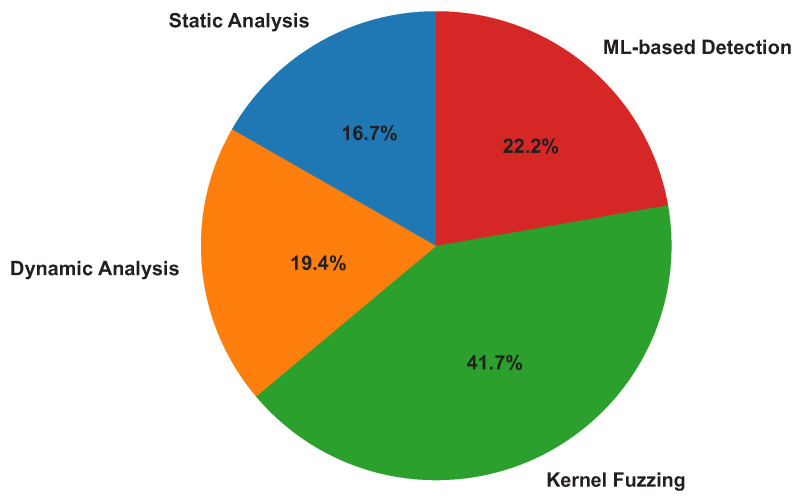
Classification of kernel vulnerability detection techniques across selected studies.

Table 6 provides a structured comparison of the principal kernel-level vulnerability detection techniques, including associated tools, artifacts, algorithms, and representative reviewed studies. The comparison indicates that fuzzing-based approaches demonstrate the strongest empirical support for the effectiveness of vulnerability discovery. Classification of kernel vulnerability detection techniques across selected studies are shown in the Figure 13.

In contrast, static analysis techniques, while valuable for early detection, show more limited empirical support because of persistent false-positive limitations and scalability challenges [9,10]. Dynamic and hypervisor-based monitoring approaches exhibit strong detection capabilities but introduce measurable performance overhead, which may constrain their deployment in real-time or resource-constrained environments [3,8].

The synthesis further indicates that kernel fuzzing dominates contemporary detection research, supported by empirical evidence demonstrating high crash discovery rates and improved code coverage.

Static analysis tools remain essential for early-stage vulnerability identification; however, they are frequently associated with elevated false-positive rates and limited scalability when applied to large and complex kernel codebases. Despite these limitations, static techniques continue to play an important role in detecting logic flaws, unsafe memory operations, and race conditions during development.

Dynamic and runtime monitoring approaches offer improved detection accuracy by analyzing kernel behavior during execution. Nevertheless, these techniques introduce computational overhead and may be vulnerable to evasion by sophisticated stealth-oriented attacks.

More recently, machine learning-based techniques have demonstrated promise, mainly as guidance and prioritization components within vulnerability discovery pipelines. However, the literature emphasizes that their reliability remains sensitive to dataset quality, kernel-version heterogeneity, feature-selection bias, and environmental drift, limiting widespread deployment in production systems [9].

Overall, the findings suggest that fuzzing-based techniques currently represent the most effective vulnerability discovery mechanism, while hybrid approaches integrating static, dynamic, and learning-based methods are increasingly advocated for to address detection blind spots.

### 4.3. RQ3: Kernel Vulnerability Mitigation and Exploit Prevention

**Figure 14:**
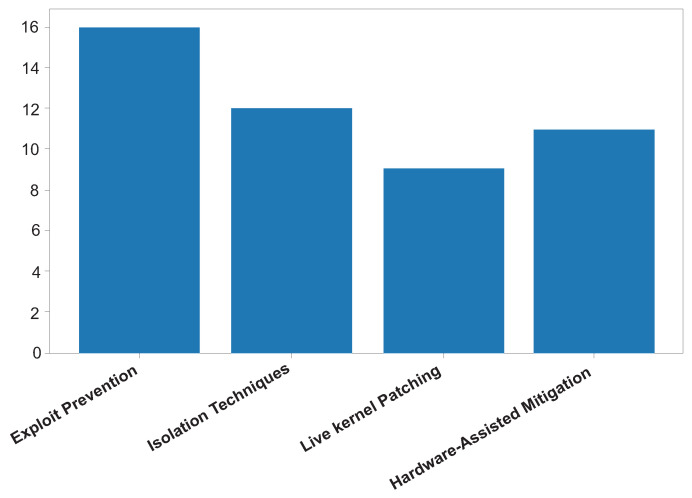
Kernel vulnerability mitigation strategies reported in the literature.

Figure 14 shows the Kernel vulnerability mitigation strategies reported in the literature. The reviewed studies shown in Table 7 indicate that kernel-level mitigation strategies primarily aim to limit exploit success, reduce attack surfaces, and contain post-exploitation impact rather than eliminate vulnerabilities entirely. These mechanisms form a critical defensive layer designed to increase attacker effort and disrupt exploitation chains.

The most commonly reported mitigation techniques include the following:

- Exploit prevention mechanisms (e.g., ASLR and DEP);
- Privilege separation and driver isolation;
- Live kernel patching;
- Hardware-assisted protections (e.g., Intel CET, and ARM TrustZone).

Exploit prevention mechanisms such as Address Space Layout Randomization (ASLR) and Data Execution Prevention (DEP) are widely implemented across modern OSs. The literature consistently highlights their effectiveness in mitigating memory corruption and code-injection attacks by randomizing memory layouts and preventing execution of non-executable regions [2,4]. However, several studies report that advanced techniques, including return-oriented programming (ROP) and side channel-assisted exploits, can partially bypass these defenses.

Privilege separation and driver isolation mechanisms focus on reducing the kernel attack surface by limiting direct access to critical kernel components. By isolating drivers and restricting privilege domains, these strategies significantly decrease the likelihood of full kernel compromise [13,14]. Nevertheless, the reviewed research notes that increased architectural complexity and compatibility constraints may affect practical deployment.

Live kernel patching emerges in the reviewed literature as an important operational mitigation because it enables vulnerability remediation without a system reboot, thereby maintaining service availability while improving security posture. Despite its advantages, challenges related to patch validation, dependency conflicts, and runtime stability remain prominent concerns.

Hardware-assisted protections provide an additional resilience layer by enforcing control-flow integrity, memory isolation, and secure execution environments [1,2]. While these mechanisms strengthen defenses against sophisticated attacks, the literature acknowledges persistent exposure to microarchitectural and side-channel threats that hardware safeguards alone cannot fully mitigate.

Overall, the findings suggest that effective kernel security relies on defense-in-depth strategies combining software-based mitigations, architectural isolation, and hardware-assisted protections. No single mitigation technique offers comprehensive protection against evolving kernel exploitation methods.

## 5. Discussion

### 5.1. Limitations of the Study

Despite providing a comprehensive synthesis of kernel-level security mechanisms, vulnerability detection techniques, and mitigation strategies, this review is subject to several limitations.

First, the scope of the study was constrained by the selected academic databases: Taylor & Francis Online, ACM Digital Library, ScienceDirect, Sage Journals, and IEEE Xplore. Therefore, relevant studies published in less accessible venues may have been omitted, including workshop papers, industrial evaluations, or security analyses not indexed by these databases. Consequently, some recent advancements in kernel security may be under-represented.

Second, the reviewed literature exhibits a strong bias toward Linux-based operating systems [7,12,13,14,17]. While Linux dominates academic research because of its accessibility and open-source ecosystem, comparatively fewer peer-reviewed studies investigate Windows, macOS, or real-time operating systems. This imbalance limits the generalizability of the findings across heterogeneous kernel architectures.

Third, inconsistencies in reported performance evaluation metrics restricted quantitative comparison across studies. Measures such as computational overhead, memory consumption, detection latency, and scalability were not uniformly defined or assessed [8,9]. As a result, the synthesis relied primarily on qualitative interpretation rather than statistical aggregation.

Finally, the rapidly evolving nature of kernel security presents an inherent temporal limitation. Kernel architectures, attack techniques, and defense mechanisms continuously change, which may reduce the long-term stability of some conclusions. Findings that are valid under current threat models may require reassessment as new vulnerabilities and mitigation technologies emerge.

Although Table 4 indicates that all included studies met the minimum quality criteria, the binary scoring model may still oversimplify methodological nuances. Variations in experimental rigor, dataset characteristics, and validation depth are not fully captured within the screening framework, which is why the final interpretation is based on narrative synthesis rather than numeric ranking.

### 5.2. Future Research Directions

The reviewed studies suggest several concrete directions for future work. First, more cross-platform kernel-security evaluations are needed so that Linux-centric findings can be compared against Windows, macOS, and microkernel-based systems under consistent assumptions. Second, the field would benefit from standardized evaluation metrics for overhead, detection fidelity, exploit resistance, and deployment complexity, which would improve comparability across studies [8,9].

Third, learning-assisted kernel security requires more rigorous validation across kernel versions, workloads, and hardware profiles before it can be treated as a dependable production defense [9]. Fourth, hardware-assisted protection should be studied together with software hardening because the reviewed evidence shows that neither layer is sufficient on its own against advanced exploit chains [1,2]. Finally, stronger kernel-level isolation is still needed for containerized, virtualized, and cloud-native deployments, where shared kernels amplify the impact of isolation failures [13,14].

## 6. Conclusions

This systematic review synthesized 30 peer-reviewed studies published between 2010 and 2024 to examine kernel-level security mechanisms, vulnerability detection techniques, and mitigation strategies in modern operating systems. The review shows that effective kernel protection is built around layered defenses combining policy enforcement; memory isolation; integrity verification; exploit mitigation; and, increasingly, hardware-assisted protection.

Across the reviewed literature, mandatory access control and memory protection techniques remain the most established protection layers, while guided fuzzing is the most consistently effective vulnerability discovery method. Runtime integrity monitoring and hardware-assisted defenses provide important complementary value, but they introduce deployment, performance, and portability tradeoffs. The synthesis also shows that the evidence base is still heavily Linux-centric, which limits broader cross-platform conclusions.

Overall, the findings support a defense-in-depth view of kernel security: no single mechanism is sufficient on its own, and future progress depends on the integration of software hardening, operational mitigation, and hardware-aware isolation under more rigorous and comparable evaluation practices.

## Figures and Tables

**Table 1:** Mapping of research questions to objectives and motivations.

RQ	RQ Statement	Motivation
RQ 1	What kernel-level security mechanisms exist in modern OSs?	To identify core protection techniques.
RQ 1.1	How do access control frameworks improve kernel security?	Access control is essential for privilege enforcement.
RQ 1.2	What memory protection mechanisms prevent kernel exploitation?	Memory corruption is a major source of kernel attacks.
RQ 1.3	How effective are hardware-assisted security features?	Hardware support enhances kernel isolation.
RQ 2	What techniques detect kernel vulnerabilities?	Early detection reduces attack success.
RQ 2.1	What static analysis tools exist for kernel vulnerability detection?	Static tools help detect bugs before execution.
RQ 2.2	What dynamic analysis methods exist?	Runtime tools detect real-time exploit attempts.
RQ 3	What mitigation strategies exist for kernel vulnerabilities?	To evaluate how OS kernels prevent exploitation.
RQ 3.1	How effective are exploit mitigation tools?	Understanding effectiveness improves defenses.
RQ 3.2	What limitations exist in current mitigation techniques?	Identifying weaknesses helps future improvement.
RQ 3.3	What enhancements are proposed?	Guides future research and OS design.

**Table 2:** Database-specific search strings used in the review. The phrasing varies slightly across databases because of platform-specific query syntax and search-engine behavior. In particular, the ACM Digital Library query uses a broader OR-based structure to avoid overly restrictive retrieval caused by strict phrase matching in the ACM interface. The same inclusion and screening criteria were subsequently applied to all retrieved records.

Database	Search String
Taylor & Francis Online	(“Kernel Security” OR “Kernel-Level Security”) AND (“Vulnerability Detection” OR “Exploit Mitigation”) AND (“Operating Systems”)
ACM Digital Library	(“Kernel Security” AND “Vulnerability Detection”) OR (“Kernel Exploitation Mitigation”) OR (“Kernel Fuzzing”)
Science Direct	(“Operating System Kernel” AND “Security Mechanisms”) AND (“Vulnerability Detection” OR “Mitigation Techniques”)
Sage Journals	(“Kernel Security” OR “Kernel-Level Security”) AND (“Operating Systems”) AND (“Vulnerability Detection” OR “Mitigation”)
IEEE Xplore	(“Kernel Security” AND “Exploit Mitigation”) OR (“Kernel Integrity” AND “Vulnerability Detection”)

**Table 3:** Selection and Screening Process.

Phase	Process	Selection Criteria	Taylor & Francis Online	ACM Digital Library	Science Direct	Sage	IEEE Xplore	Total
1	Searching	Keywords	120	140	160	90	170	680
2	Screening	Title	65	75	90	50	95	375
3	Further screening	Abstract	38	45	52	30	55	220
4	Further screening	Introduction, and Conclusion	18	22	24	14	20	98
5	Evaluation	Complete Article	6	7	7	5	5	30

**Table 4:** Binary CASP-based quality screening of the included studies.

Study ID	Reference	Clear Objectives	Valid Methodology	Kernel Security Focus	Experimental Validation	Contribution	Total Score
S1	[18]	1	1	1	1	1	5
S2	[12]	1	1	1	1	0	4
S3	[1]	1	1	1	0	1	4
S4	[19]	1	1	1	1	1	5
S5	[2]	1	0	1	1	1	4
S6	[20]	1	1	1	1	1	5
S7	[7]	1	1	1	0	1	4
S8	[17]	1	1	0	1	1	4
S9	[21]	1	1	1	1	1	5
S10	[22]	1	1	1	1	0	4
S11	[8]	1	1	1	1	1	5
S12	[13]	1	0	1	1	1	4
S13	[14]	1	1	1	0	1	4
S14	[9]	1	1	1	1	1	5
S15	[23]	1	1	0	1	1	4
S16	[24]	1	1	1	1	1	5
S17	[25]	1	1	1	0	1	4
S18	[26]	1	0	1	1	1	4
S19	[27]	1	1	1	1	1	5
S20	[3]	1	1	1	1	0	4
S21	[28]	1	1	1	1	1	5
S22	[29]	1	0	1	1	1	4
S23	[4]	1	1	1	0	1	4
S24	[30]	1	1	1	1	1	5
S25	[31]	1	1	0	1	1	4
S26	[32]	1	1	1	1	1	5
S27	[10]	1	1	1	0	1	4
S28	[33]	1	0	1	1	1	4
S29	[34]	1	1	1	1	1	5
S30	[35]	1	1	1	1	0	4

**Table 5:** Summary of selected references.

Reference	Publication Year	Publication Medium	OS/Scope	Tools/Techniques	Main Focus
[3]	2011	Conference	Commodity OS kernels	Hypervisor-based protection	Kernel integrity protection against untrusted extensions
[10]	2012	Journal	OS security overview	Survey synthesis	Broad taxonomy of operating-system security research
[17]	2020	Journal	Linux/cloud systems	KASLR-MT	Kernel address-space randomization in multi-tenant environments
[8]	2020	Journal	Embedded/IoT kernels	Trusted-execution runtime integrity protection	Runtime kernel integrity assurance
[7]	2021	Conference	Linux kernel fuzzing	Relation learning-guided fuzzing	Improved kernel bug discovery through guided fuzzing
[12]	2022	Conference	Linux kernel	Attribute-based access control	Fine-grained policy enforcement inside the kernel
[1]	2022	Conference	Linux drivers	Continuous re-randomization	Hardware-aware address-space diversification
[13]	2022	Journal	Containerized Linux systems	LSM and cgroup-based controls	Kernel-mediated isolation for sensitive application data
[14]	2023	Journal	Monolithic kernels	Intra-kernel sandboxing	Attack-surface reduction and robustness improvement
[9]	2024	Journal	Vulnerability discovery methods	Machine learning-assisted fuzzing review	Systematic synthesis of learning-based vulnerability detection

**Table 6:** Vulnerability detection techniques.

Title	Objects/Tools/Artifacts	Protocols	Algorithms	Assessment Focus	Empirical Evidence	Representative Reviewed Studies
Syzkaller Kernel Fuzzing	Linux Kernel	Syscall Interface	Random + Guided Mutation	Crash discovery and coverage	Strong	[7,9]
Hybrid Kernel Fuzzing	Kernel APIs	Hybrid Fuzzing	ML-guided Fuzzing	Bug-discovery efficiency	Strong	[7,9]
Static Kernel Analysis	Kernel Source Code	Code Analysis	Symbolic Execution	False positives and scalability	Moderate	[9,10]
Runtime Integrity Monitoring	Kernel Memory	DFIM	Integrity Checks	Detection fidelity and overhead	Strong	[3,8]
ML-based Detection	Input prioritization and vulnerability discovery	Learning-assisted analysis	Guided classification and ranking	Precision, recall, and generalization	Moderate	[9]
Hypervisor-based Monitoring	Kernel Execution	VM Isolation	Monitoring Hooks	Security gain versus overhead	Strong	[3]

**Table 7:** Kernel vulnerability mitigation strategies.

Mitigation Category	Security Objective	Representative Reviewed Studies	Reported Strengths	Reported Limitations	Implication
Memory-execution controls	Disrupt code injection and code reuse	[2,4]	Increase attacker effort and reduce exploit reliability	Can be weakened by leaks, ROP, and side channels	Best used with complementary isolation controls
Privilege separation and driver isolation	Reduce the kernel attack surface	[13,14]	Contain faults and limit lateral privilege abuse	Architectural complexity and compatibility overhead	Valuable in modular and containerized deployments
Runtime integrity protection	Detect unauthorized kernel modification	[3,8]	Strong post-deployment monitoring and tamper resistance	Runtime cost and dependency on trusted components	Complements preventive hardening rather than replacing it
Hardware-assisted isolation	Enforce stronger execution and memory boundaries	[1]	Improves resistance to advanced attack chains	Platform dependence and residual microarchitectural risk	Important for future high-assurance kernel designs
Operational mitigation and patching	Remediate vulnerabilities while preserving availability	[3,14]	Improves resilience during maintenance windows	Validation, dependency, and rollback challenges	Requires disciplined operational testing

## Data Availability

To enhance transparency and reproducibility, the data and supplementary study-traceability materials associated with this work are publicly available at https://github.com/drzeeshanaliofficial/A-Systematic-Review-of-Kernel-Level-Security-Mechanism (accessed on 5 April 2026).

## Abbreviations

The following abbreviations are used in this manuscript:

MAC	Mandatory Access Control
PRISMA	Preferred Reporting Items for Systematic Reviews and Meta-Analyses
CASP	Critical Appraisal Skills Programme
OS	Operating System
RQ	Research Question
ASLR	Address Space Layout Randomization
DEP	Data Execution Prevention
KASLR	Kernel Address Space Layout Randomization
SMEP	Supervisor Mode Execution Prevention
SMAP	Supervisor Mode Access Prevention
CVE	Common Vulnerabilities and Exposures
ML	Machine Learning
AI	Artificial Intelligence
ABAC	Attribute-Based Access Control
LSM	Linux Security Module
SGX	Software Guard Extension
CET	Control-flow Enforcement Technology
SEV	Secure Encrypted Virtualization
TEE	Trusted Execution Environment
VM	Virtual Machine
IoT	Internet of Things
IIoT	Industrial Internet of Things
ACM	Association for Computing Machinery
IEEE	Institute of Electrical and Electronics Engineers
